# Study on Hypoglycemic Effect of the Drug Pair of Astragalus Radix and Dioscoreae Rhizoma in T2DM Rats by Network Pharmacology and Metabonomics

**DOI:** 10.3390/molecules24224050

**Published:** 2019-11-08

**Authors:** Qian Guo, Wanlin Niu, Xuejia Li, Hongru Guo, Na Zhang, Xiufeng Wang, Lirong Wu

**Affiliations:** 1 Guangdong Provincial Key Laboratory of Pharmaceutical Bioactive Substances, Guangdong Pharmaceutical University, Guangzhou 510006, Guangdong, China; GQgdpu@outlook.com (Q.G.); 945419778@163.com (W.N.); LXJgdpu@outlook.com (X.L.); 2 School of Biosciences and Biopharmaceutics, Guangdong Pharmaceutical University, Guangzhou 510006, Guangdong, China; 3 School of Pharmacy, Guangdong Pharmaceutical University, Guangzhou 510006, Guangdong, China; ZNgdpu@outlook.com; 4 School of Traditional Chinese Medicine, Guangdong Pharmaceutical University, Guangzhou 510006, Guangdong, China; GHRgdpu@outlook.com; 5 College of Medical Information Engineering, Guangdong Pharmaceutical University, Guangzhou 510006, Guangdong, China

**Keywords:** Dioscoreae Rhizomacomes, Astragalus Radix, type 2 diabetes, metabolomics, network pharmacology

## Abstract

Type 2 diabetes mellitus (T2DM) is a metabolic disease accompanied by a series of diseases such as diabetic nephropathy. The drug pair (HS) of Astragalus Radix (HQ) and Dioscoreae Rhizoma (SY) was designed by Dr. Shi Jinmo to improve the treatment of T2DM. However, the exact mechanism involved requires further clarification. In this work, ^1^H-NMR–based metabonomics and network pharmacology were adopted. Metabolic profiling indicated that the metabolic perturbation was reduced after HS treatment. The results found 21 biomarkers. According to the network pharmacology, we found that the regulation of T2DM was primarily associated with 18 active compounds in HS. These active compounds mainly had an effect on 135 targets. Subsequently, combining network pharmacology and metabonomics, we found four target proteins, which indicated that HS has potential hypoglycemic effects through regulating monoamine oxidases B (MAOB), acetyl-CoA carboxylase 1 (ACACA), carbonic anhydrase 2 (CA2), and catalase (CAT). In conclusion, the result showed that these four targets might be the most relevant targets for the treatment of T2DM with HS. This study clarified the mechanism of HS in the treatment of T2DM and also confirmed the feasibility of combining metabonomics and network pharmacology to study the mechanisms of traditional Chinese medicine (TCM). In the future, this approach may be a potentially powerful tool to discovery active components of traditional Chinese medicines and elucidate their mechanisms.

## 1. Introduction

Type 2 diabetes mellitus (T2DM) is a common metabolic syndrome caused by the inability of pancreatic β cells to produce enough insulin or the body’s inability to use insulin effectively. The World Health Organization (WHO) estimates that the number of people with diabetes in the world will increase to 592 million by 2035 [1]. A large number of proposals and hypothesis have been developed to describe its mechanisms. β-cell dysfunction, endoplasmic reticulum stress (ER-stress) in b-cells, tissue lipid accumulation, oxidative stress, tissue inflammation, and some signaling pathway such as JNK/SAPK, p38 MAPK, and Wnt are the most commonly known factors linked to T2DM [2,3,4]. Once T2DM occurs, it imparts long-term consequences that may include atherosclerosis, neuropathy, retinopathy, and nephropathy [5]. As a result, effective control of blood glucose level is very important to prevent diabetes complications and improve the health of T2DM patients. At present, insulin, metformin, α-glucosidase inhibitors, thiazolidinediones, glucagonlike peptide–based therapy, dipeptidyl peptidase 4 inhibitors, and sodium-glucose transport protein 2 (SGLT-2) inhibitors have been clinical used in the treatment of T2DM [5]. However, they can result in many adverse reactions, such as hypoglycemia, gastrointestinal discomfort, and so on [6]. Thus, there is a general trend to seek the treatment of T2DM from traditional Chinese medicine (TCM) or natural products with stable curative effect and few side effects.

According to traditional Chinese medicine (TCM), Dioscoreae Rhizoma (SY) is sweet and natured, it can supplementing qi, nourish yin, and tonify spleen, lung, and kidney. It also can improve the level of immune regulation, anti-oxidation, anti-tumor, and hypoglycemic effects. Go et al. [7] reported that SY could promote the release of GLP-1 and improve the function of β-cells maintaining normal insulin and glucose levels in a rat model of streptozotocin-induced diabetes. According to TCM, Astragalus Radix (HQ) is sweet in taste and warm in nature, it also has the effects of invigorating Qi, replenishing the spleen, boosting Qi, securing the exterior, and inducing diuresis to alleviate edema. Its pharmacological effects include immune regulation, liver protection and hypoglycemic. Zou et al. [8] showed that HQ could alleviate glucose toxicity by increasing liver glycogen synthesis and skeletal muscle glucose translocation in the streptozotocin-induced T2DM rat model. Moreover, Dr. Shi Jinmo, a famous modern doctor, proposed the famous hypoglycemic drug pair HS (HQ and SY). He believed that HQ and SY were good for spleen yang and spleen yin, respectively [7]. According to TCM, their combination give consideration to both yin and yang which were beneficial to the spleen function [9]. A large number of studies have also found that the high frequency of HQ and SY appears in TCM compound or proprietary Chinese medicine in the clinical treatment of T2DM. Yan et al. [10] reported that the HQ and SY drug pair could effectively prevent and treat diabetes in multiple low doses of streptozotocin–induced diabetic mice. At the same time, our previous study [11] found that, compared with HQ and SY groups, the drug pair of HQ and SY had a more obvious hypoglycemic effect on streptozocins-induced T2DM rats. Therefore, this article uses HS (HQ and SY) to explore the mechanism of action in the treatment of T2DM. At present, the efficacy evaluation and mechanism of action of TCM are based on the method of chemical drugs, which ignore the overall effect of TCM. Hence, it is necessary to establish a method suitable for TCM.

**Figure 1:**
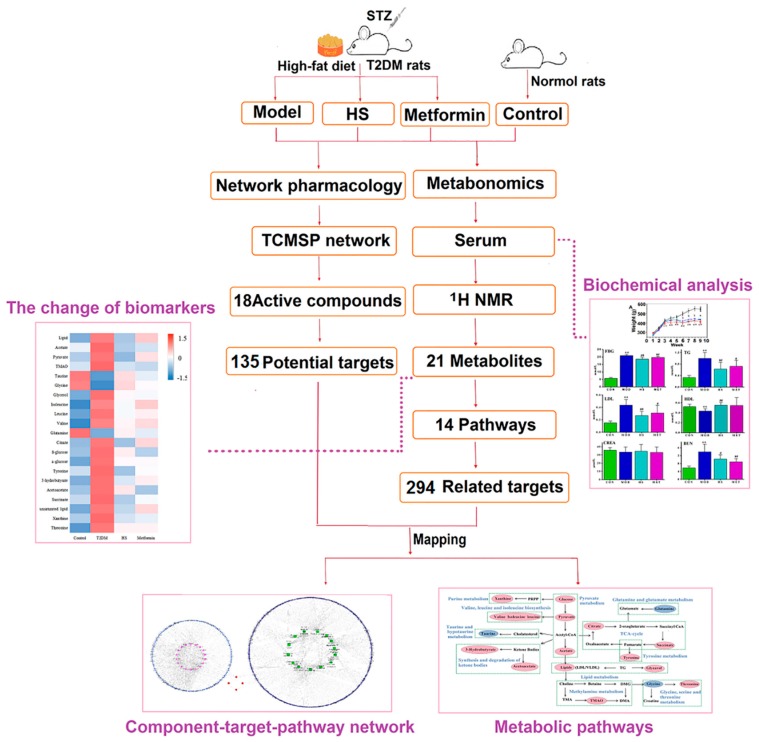
The overall scheme of the research processes.

Metabonomics reflects the changes in the metabolic network of the body under the intervention of diseases or drugs by detecting small molecular metabolites in biological samples. It can comprehensively analyze organisms from a whole perspective, which coincide with the concept of “wholism” in TCM. Nuclear magnetic resonance (NMR) has the advantages of simple sample preparation and being fast, qualitative, and quantitative. It has been widely used in metabonomics research [12]. In recent years, network pharmacology has become a powerful tool in clarifying the complex and integral mechanisms of TCM [13]. It can construct related networks from the perspective of biological systems to explore the pathogenesis of diseases and reveal the interactions between the target and the drug in vivo. In this study, the ^1^H-NMR serum metabolomics method was used to study the potential biomarkers of T2DM rats and the changes of biomarkers after HS treatment. Besides, the network of potential biomarker targets and HS treatment targets were constructed using network pharmacology. The combination of network pharmacology and metabolomics provides a new way to explain the hypoglycemic mechanism of HS in the treatment of T2DM. The overall scheme of the research process is shown in Figure 1.

## 2. Results

### 2.1. FBG, Biochemical Parameters, and Histopathological Observations

**Figure 2:**
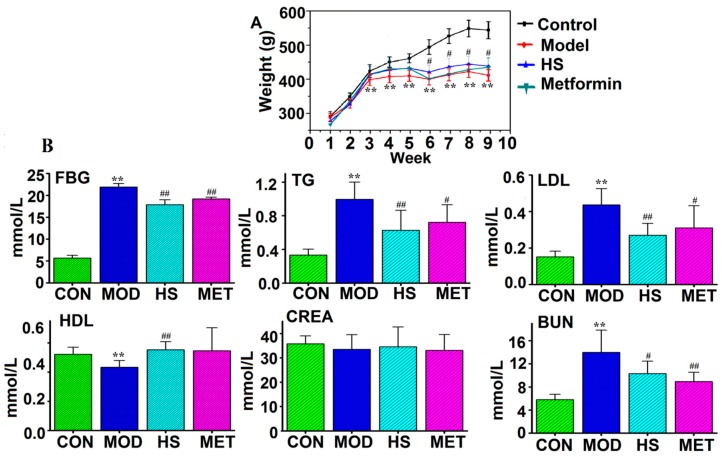
(**A**) The changes of the body weight at different time points in four groups; (**B**) The biochemical parameters used to evaluate the efficacy of the drug pair (HS) of Astragalus Radix (HQ) and Dioscoreae Rhizoma (SY) in the treatment of type 2 diabetes mellitus (T2DM). Error bars represent the mean ± SD (Student’s t-test: compared with the control group, * *p* < 0.05, ** *p* < 0.01; compared with the model group # *p* < 0.05, ## *p* < 0.01.

Figure 2A showed that, during the whole experiment, the weight of the control group gradually increased, while the model group decreased after the injection of streptozocins (week 4). This might be due to fat and protein catabolism that provides energy to cells, resulting in muscle atrophy and weight loss. After treatment with HS and metformin (week 5), there was no significant increase in body weight (*p* > 0.05), but to some extent, it could improve the trend of weight loss. Figure 2B showed that the level of FBG in the model group were significantly higher than control group, HS and metformin treatment could significantly reduce FBG in T2DM rats (*p* < 0.01). At the same time, compared with the control group, the level of TG, LDL, and BUN in the model group were significantly increased (*p* < 0.01), HDL was significantly decreased, but the level of CREA was not significantly changed (*p* > 0.05). It indicated that the lipid metabolisms of the model rats were disturbed, and their renal function might be slightly impaired. However, the changes of the above indexes could be significantly reversed after HS treatment, suggesting that HS could improve the lipid metabolism disorder of T2DM.

### 2.2. Pattern Recognition Analysis and Identification of Biomarker

**Figure 3:**
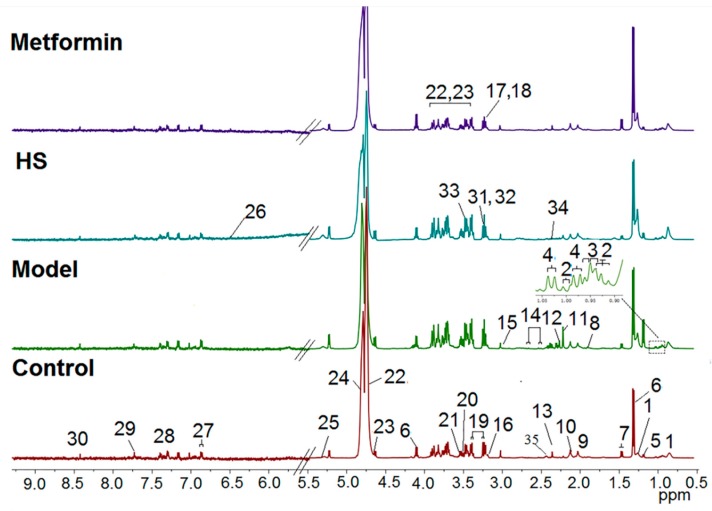
^1^H-NMR spectra of serum obtained from the 4 groups of rats. Keys: 1.lipid; 2. isoleucine; 3. leucine; 4. valine; 5. 3-hydrobutyrate; 6. lactate; 7. alanine; 8. acetate; 9. *N*-acetylglycoprotein; 10. methionine; 11. choline alfoscerate; 12. acetoacetate; 13. pyruvate; 14. citrate; 15. creatine; 16. choline; 17. phosphatidylcholine (PC); 18. glycerophosphocholine (GPC); 19. taurine; 20. glycine; 21. glycerol; 22. β-glucose; 23. α-glucose; 24. unsaturated lipid; 25. allantoin; 26. fumarate; 27. tyrosine; 28. phenylalanine; 29. xanthine; 30. formate; 31. Trimethylamine *N*-oxide (TMAO); 32. betaine; 33. threonine; 34. succinate 35. glutamine.

The representative ^1^H-NMR spectra serum samples from control, model, HS, and metformin treated groups are shown in Figure 3, and a total of 35 metabolites were identified. Due to individual differences, it was difficult to directly observe the changes of serum metabolites in the four groups. Thus, we performed multivariate data analysis to determine the metabolic markers in T2DM rats.

**Figure 4:**
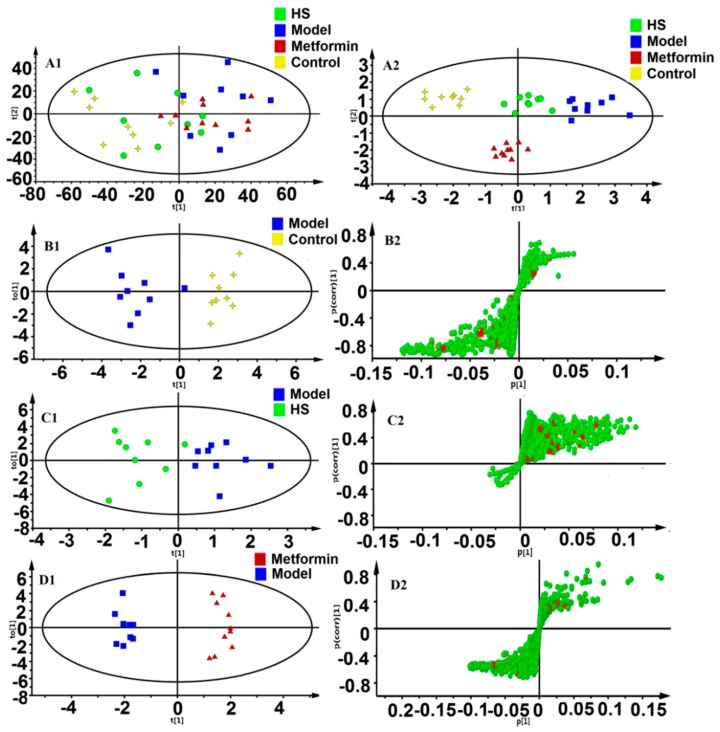
Multivariate analyses of serum ^1^H-NMR spectra data. A1: Principal Component Analysis (PCA) scores plot (R2X = 0.843), A2: orthogonal partial least squares discriminant analysis (OPLS-DA) scores plot (R2X = 0.922, R2Y = 0.851, Q2= 0.595, *p*-value = 0.015); B1, B2: OPLS-DA score plot and S-plot for control and model group (R2X = 0.711, R2Y = 0.873, Q2 = 0.734, *p*-value = 0.001); C1, C2: OPLS-DA score plot and S-plot for model and HS treatment group (R2X = 0.697, R2Y = 0.776, Q2 = 0.502, *p*-value = 0.047); D1, D2: OPLS-DA score plot and S-plot for model and metformin treatment group (R2X = 0.818, R2Y = 0.981, Q2 = 0.939, *p*-value < 0.001). In the S-plot, variables marked in red could be treated as potential biomarkers.

In order to prevent model from over-fitting, the performance of the PLS-DA models between these groups were evaluated using 200-times permutation (Figure S1). The results showed that the model established had good discriminability, adaptability, and predictive ability. Figure 4A1 showed a PCA score plot of serum, but the discriminationis of four groups were not very distinct. Thus, the OPLS-DA model was established to maximize the distinction between groups (Figure 4A2,B1,C1,D1), the CV-ANOVA was used to verify the OPLS-DA model, and the corresponding *p*-values were calculated, indicating that the OPLS-DA models for serum were effective. Meanwhile, S-plots (Figure 4B2,C2,D2) were applied to identify the potential biomarkers obtained from four groups, and the further the ions far from the origin, the higher the value of the obtained Variable Importance in the Projection (VIP).

**Figure 5:**
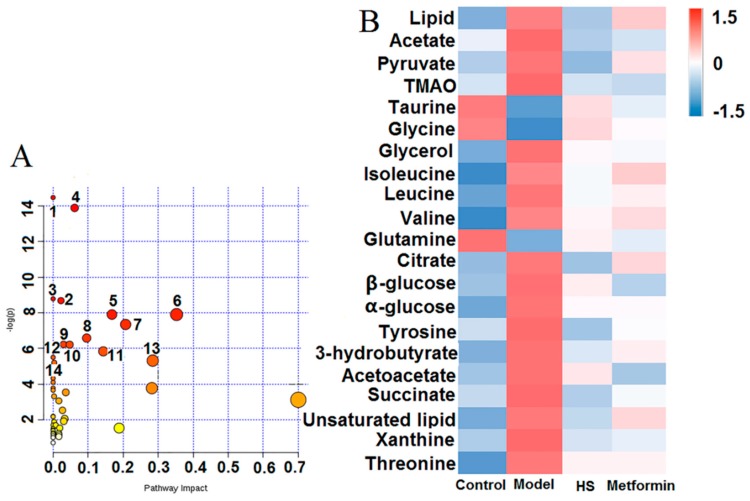
(**A**) Summary of pathway analysis with MetaboAnalyst 3.0. Each dot represents a metabolic pathway, and the label corresponds to the pathway number in Table 2; (**B**) The change of 21 biomarkers in control, model, HS and metformin rats for serum.

VIP > 1 could be considered important in a given model. Therefore, 21 potential biomarkers among groups were finally screened out on the basis of VIP > 1 and *p* < 0.05 (Table 1). As shown in Table 1 and the S-plot, compared with the control group, the levels of lipid, acetate, pyruvate, TMAO, glycerol, isoleucine, leucine, valine, citrate, β-glucose, α-glucose, tyrosine, acetoacetate,3-hydrobutyrate, succinate, unsaturated lipid, xanthine, and threonine significantly decreased in model group, while taurine, glycine, and glutamine increased significantly, and the heat map of the 21 biomarkers are shown in Figure 5B. This indicated that the metabolites of model rats had changed significantly, while the level of these metabolites could be reversed by HS and metformin treatment.

### 2.3. Metabolic Pathways and Targets Analysis

**Figure 6:**
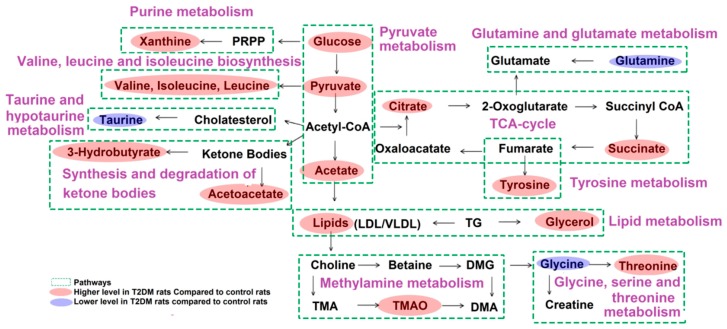
Schematic diagram of the metabolic pathways. Compared with the control group, red and blue metabolites represent increased and decreased levels, respectively.

Based on the 21 identified potential biomarkers of T2DM rats, the relevant metabolic pathways were assigned using MetaboAnalyst 3.0. The results showed that 21 biomarkers corresponded to 44 pathways, of which 14 pathways were *p* < 0.01. Those pathways were considered the most relevant pathways involved in T2DM. The results of pathway analysis were presented in Figure 5A as well as in a detailed table in Table 2. Figure 6 illustrated the key metabolic pathways during the onset and development of T2DM based on the KEGG database.

### 2.4. Network Construction

**Figure 7:**
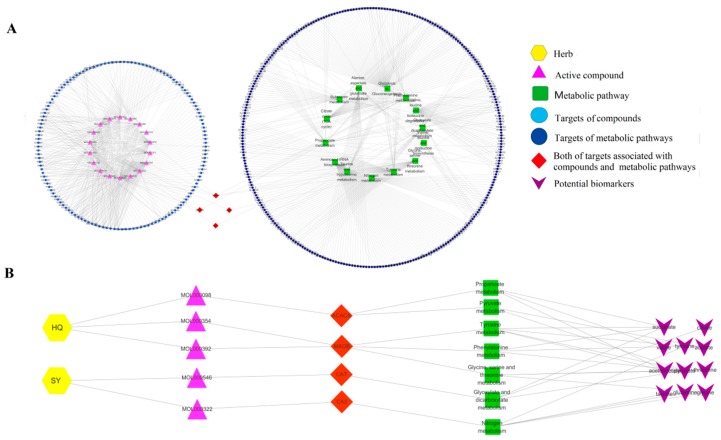
(**A**) Active component–target–pathway interaction network. (**B**) Active component–corresponding target–metabolic pathway–potential biomarker interaction network. The pink nodes represent the chemical components of HS, the azure nodes represent the predicted targets of component, the green nodes represent the metabolic pathway, and the mazarine nodes represent the target of pathway. The red nodes represent both of targets associated with component and metabolic pathway. The purple nodes represent related potential biomarker.

The results of compounds and corresponding targets were correlated with the metabolic pathway and corresponding targets. Meanwhile, the pathways were used to construct a phytochemical component–target–pathway interaction network, which was visualized by Cytoscape. As shown in Figure 7A, 18 potentially active compounds were screened out from HQ (12) and SY (6). Here, 135 predicted potential targets of HS were identified by network pharmacology. The experimental results showed that there were 21 potential biomarkers, mainly involving 14 metabolic pathways and 294 related targets. In order to further reveal the potential active components and corresponding targets in the hypoglycemic mechanism of HS, an interaction network of active components–corresponding targets–metabolic pathways–potential biomarkers was constructed based on the experimental results of metabolomics and network pharmacology. As shown in Figure 7B, four targets were considered as potential therapeutic targets, including ACACA Acetyl-CoA carboxylase 1 (ACACA), Monoamine oxidases B (MAOB), Catalase (CAT), and Carbonic anhydrase 2 (CA2).

## 3. Discussion

In the past decade, metabolomics has made significant advances in helping to understand the pathogenesis of many metabolic diseases, such as T2DM, obesity, and cancer. Obesity and T2DM are rapidly becoming common diseases caused by complex interactions between genetic, hormonal deficiencies, metabolism, and environmental factors (diet and lifestyle). The efficacy of TCM in the treatment of T2DM has been confirmed through several hundred years’ practice. In this study, the ^1^H-NMR spectra of serum samples provided the dynamic changes of endogenous small molecule metabolites, which might provide us with some important information about the mechanism of HS in the treatment of T2DM. Combined network pharmacology and serum metabonomics to find out the potential therapeutic targets, and the main targets of HS in the treatment of T2DM were studied.

T2DM is a metabolic disorder characterized by hyperglycemia and hyperlipidemia. In this study, the level of glucose in the model rats increased and the serum glucose content of the rats significantly decreased after the treatment with HS and metformin, indicating that HS had a good hypoglycemic effect, which was equivalent to 200 mg/kg metformin. Compared with the control group, the level of lipid, TG and LDL in T2DM rats were increased significantly, the level of HDL was decreased significantly. Meanwhile, as the intermediate products of lipid metabolism, the level of 3-hydrobutyrate, acetate and glycerol increased significantly in the model rats [14], indicating that the ketone body metabolism and β-oxidation of fatty acids were enhanced in the model rats under the high glucose environment [15]. Interestingly, we found that the above indexes were significantly corrected after HS treatment in the model rats, suggesting that HS might regulate the disorder of lipid metabolism in rats to some extent.

Carbohydrate metabolism is an important metabolic pathway to regulate energy productions in the human body [16]. Abnormal accumulation of glycolytic intermediates may reflect the disorder of energy metabolism in T2DM patients. Hyperglycemia could increase the rate of glucose oxidation leads to the excessive formation of pyruvate. Meanwhile, the enhancement of glycolytic pathway would affect the metabolism of intermediate in TCA cycle [17,18,19]. The results of this study showed that the content of pyruvate and TCA cycle intermediates (citrate and succinate) in the model group were significantly higher than the control group, indicating that the metabolic adaptation of T2DM rats were directed towards glycolytic pathways in the progression of T2DM. While, after HS treatment, the level of pyruvate, citrate and succinate in the model rats were decreased significantly, indicating that HS might regulate the disturbance of energy metabolism in T2DM rats. Acetate is one of the intermediate products of glycolytic, which is produced by the liver and partially involved in the metabolism of lipids and carbohydrates and absorbed through the intestinal tract [20]. The experimental results showed that the level of acetate was augmented in T2DM rats, indicating that the acetyl-CoA synthetase enzyme was inhibited, which was responsible for the conversion of acetate into acetyl-CoA. On the other hand, the increase of acetate level might be a sign of lipid accumulation [21].

Amino acids play an important role in many metabolic pathways. Many studies had showed that T2DM would result in aberrant amino acid metabolism [22]. Essential amino acids such as branched-chain amino acids (BCAAs), including valine, leucine, and isoleucine, are required by the human body through diet for many major metabolic processes [23]. The degradation of BCAA metabolism was linked with insulin sensitivity [24]. Increased BCAA levels were also associated with decreased activity of key BCAA catabolic enzymes in liver and adipose tissue or increased muscle protein degradation [25]. In summary, BCAAs level were affected by insulin resistance, protein catabolism, gluconeogenesis, renal protein synthesis, and other factors. The results of this study also confirmed this point. The serum BCAAs level of T2DM rats were increased, and HS treatment could significantly reduce the level of BCAAs, indicating that HS could treat T2DM by reducing insulin resistance, improving insulin sensitivity, and regulating protein metabolism. Glutamine is a non-essential amino acid synthesized by the body [26]. Many studies indicated that glutamine supplementation could enhance insulin release from islet β-cells and transport of glucose transporter 4 (GLUT4) [27]. Decreased serum glutamine levels in obese people may increase the risk of T2DM [28]. This was consistent with our experimental results. The serum level of glutamine in the model group was decreased, and the level of glutamine was significantly increased after treatment with HS, suggesting that HS might improve the disorder of glucose metabolism in T2DM rats by regulating the content of glutamine. Glycine changes significantly in metabolic disorders. Considerable studies [29] had shown that the relative concentration of glycine was decreased in the serum of diabetic patients compare to the control, and its concentration was negatively correlated with T2DM [30]. In this study, compared with the control group, the down regulation of glycine in T2DM rats might be related to lipid oxidation and oxidative stress. The glycine level was up regulated after HS treatment, indicating that the oxidative stress response could be alleviated after HS treatment. Nevertheless, several other articles had reported the level of serum glycine was increased in T2DM patients. In fact, the exact effects of glycine fluctuation in various biological fluids still remain vague. Overall, these findings reveal that the disturbance of the amino acid in these metabolic diseases may involve complex biological processes, depending on the severity of the disease [31]. Taurine is an intermediate metabolite of bile acid metabolism, which is mainly excreted by the kidney. Taurine also plays an important role in antioxidant activity and maintain the structural integrity of cell membranes [32]. Modern studies had also found that taurine could play a hypoglycemic role through gluconeogenesis [33]. In this study, the level of taurine in the T2DM group decreased, while increased significantly after HS treatment, indicating that HS played a certain role in controlling the occurrence and development of T2DM through taurine and taurine metabolic pathways.

Intestinal microbiomes are directly participated in the metabolism of dietary lecithin to generate trimethylamine *N*-oxide (TMAO). Therefore, the concentration of TMOA could be used as an indicator of intestinal microbial status [34]. In this study, it was found that TMAO level in serum of T2DM rats were significantly higher than that of normal rats, indicating that the intestinal functions of model rats were disturbed. However, HS treatment could decrease the TMAO level, as HS treatment could improve the status of intestinal microflora. However, some of the results were inconsistent with our results on the level of TMAO [35]. These discrepancies might be caused by the differences in dietary composition. Altogether, the fluctuations in TMAO content could indicate the importance of metabolites produced by intestinal microbiomes in the regulation of T2DM. Xanthine is an intermediate of adenosine metabolism. As a substrate of xanthine oxidase, it could induce oxidative stress by enhancing superoxide molecules [36]. Abnormal accumulation of ROS within the cell would affect microvascular function and cause tissue damage. A lot of evidence has verified that ROS is one of the most important pathogenesis of T2DM [37,38]. In this study, the level of xanthine increased in T2DM rats and decreased in HS rats, indicating that HS protected the body from oxidative stress by scavenging free radicals.

Acetyl-CoA carboxylase (ACC) has two subtypes, ACC1 (ACCα) and ACC2 (ACCβ), that are regulated and expressed by ACACA and ACACB [39]. ACACA is mainly expressed in adipose tissue (liver, adipose tissue, mammary glands) and is responsible for long-chain fatty acid synthesis [40]. ACC is one of the rate-limiting enzymes for fatty acid synthesis in the body, which could catalyz acetyl-CoA to form malonyl-CoA [41]. However, high levels of malonyl-CoA could reduce fatty acid oxidation and increase fatty acid synthesis, leading to accumulation of fatty acids in the body. At present, ACC inhibitors, as a new treatment, could be used to treat various metabolic disorders, which have gradually aroused extensive attention. In recent years, a large number of studies have shown that the level of oxidative stress in the body is closely related to diabetes. Antioxidant defense systems could protect the body from oxidative stress by clearing free radicals and maintaining reactive oxygen species (ROS) at the physiological level [42]. The primary scavenger enzymes in mammals include superoxide dismutase (SOD), catalase (CAT), and glutathione peroxidase (GPX), which may be involved in detoxifying ROS [43]. Among them, CAT can catalyze hydrogen peroxide and decompose into less-reactive gaseous oxygen and water molecules, thus removing the damage of the active oxygen to the cells [44]. Carbonic anhydrase (CA) is a pH-regulatory Zn metalloenzyme that rapidly catalyzes the hydration of carbon dioxide (CO2) to form bicarbonate (HCO3-) and reversible dehydration. In mammals, at least 13 isozymes of CA and CA-related proteins had been identified. Therefore, CA-2 has the widest distribution and the highest catalytic activity in the body [45,46]. When it is deficient, it can cause physiological diseases such as obstacles to growth development, kidney function, and bone absorption. Ghosh et al. [47] found that the glycosylation played a vital role in reduction of CA activity in individuals with T2DM. He suggested that the changes in CA activities in erythrocytes might signify the onset of altered metabolism in T2DM. Research showed that monoamines directly affected hormone secretion by binding to specific receptors [48]. Monoamine oxidase (MAO) is bound to the outer membrane of mitochondria and participated in the metabolism of monoamines [49]. Ganic et al. [50]. found that the inhibition of MAOA and MAOB activity might lead to decreased glucose content and stimulate insulin secretion, and the loss of MAO expression might lead to β cell dysfunction in T2DM patients.

## 4. Materials and Methods

### 4.1. Reagents and Instruments

*Astragalus membranaceus* (Fisch.) Bge. var (HQ batch number: 20171203) and *Dioscorea opposita* Thunb (SY batch number: 18Z0401) were obtained from Caizhiling Chinese Herbal Medicine Co., Ltd. (Guangdong, China) and authenticated by associate professor Hong-Yan Ma of College of Traditional Chinese Medicine of Guangdong Pharmaceutical University (Guangzhou, China). Metformin Hydrochloride Tablets were obtained from Bristol-Myers Squibb (New York, NY, USA). Streptozocins was purchased from Sigma (St. Louis, MO, USA). A Roche blood glucose meter and glucose test strips were purchased from F. Hoffmann-La Roche AG (Basel, Switzerland). High-fat diet (18% lard, 20% sucrose, 3% egg yolk, 59% basic feed) was processed by Guangdong Medical Experimental Animal Center (Guangdong, China), License No: SCXK (YUE) 2018-0002. Deuterium Oxide (D_2_O) and 5 mm nuclear magnetic resonance (NMR) tubes were obtained from Qingdao Teng Long Bio-technology Company (Qingdao, China). A 500 MHz Bruker AVANCE III NMR instrument (Billerica, MA, USA) was provided by the Center Laboratory of Guangdong Pharmaceutical University (Guangzhou, China). An automatic biochemistry analyzer and other biochemical assay kits were provided by Mindray Biomedical electronics Co., LTD (Shenzhen, China). Desktop high-speed refrigeration centrifuges (Hamburg, Germany) were provided by Guangdong Provincial Key Laboratory of Pharmaceutical Bioactive Substances (Guangdong, China).

### 4.2. Preparation of HQ and SY

First, 45 g HQ and 45 g SY were placed in a 1000 mL round bottom flask, soaked in 600 mL distilled water for 0.5 h, heated to reflux for 2 h, and then filtrated with gauze. The drug residue was boiled in the same way to get the water extract, mixed with two parts of the water extract, evaporated to 3.0 g/mL in a vacuum rotary evaporator, and stored at 4 °C until use. The gavage dose of rats was 6.3 mL/kg/day.

### 4.3. Animal Care and Experiments

Forty-six male Sprague-Dawley (SD) rats (320 ± 20 g) were provided by the Guangdong Provincial Experimental Animals Center (License no. SCXK (Yue) 2013-0002) (Guangdong, China) and kept in SPF-grade experimental animal houses. All rats were housed in a temperature-controlled room (25 ± 2 °C, 50 ± 5% humidity) with free access to food and water under a 12 h light/dark cycle. All rats were acclimatized to the new environment for one week prior to experimentation. Animal care and experimental protocols were approved by the institutional ethics committee of Guangdong Pharmaceutical University, and the batch number was SPF2017087.

All rats were randomly divided into two groups: control group (10 rats) and T2DM group (36 rats). The rats of control group were given normal diet. The T2DM group were fed with high-fat diet lasting for four weeks. After four weeks, all rats fasted but had free access to water for 12 h. The T2DM rats were injected intraperitoneally with streptozocins (35mg/kg body weight), which was configured with citrate buffer (0.1 M, pH 4.4). Meanwhile, the model rats were injected with the same volume of citrate buffer. On the third and sixth days after streptozocins injection, the level of fasting 8 h blood glucose (FBG) was measured in each group, and rats with FBG ≥ 16.7 were considered as T2DM rats. In the T2DM group, a total of 30 rats were successfully modeled, with a success rate of 83%. All the T2DM rats were randomly divided into three groups—model group (10 rats), HS treatment group (10 rats), and metformin treatment group (10 rats). The rats in HS group were given HQ and SY extracting solutions (6.3 g/kg) twice a day for four weeks. The metformin group rats were given metformin (200 mg/kg) once a day for four weeks. Control group and model group were given the same volume of distilled water. During the whole experiment, one rat in the model group and one rat in the HS group died of diabetic ketosis, and one rat in the control group died of brawl.

Simultaneously, all rats were sacrificed 12 h after the last drug administration at the eighth week. They were anesthetized with pentobarbital sodium, and blood samples were taken from the abdominal aorta. Serum of all rats was isolated by centrifugation for 15 min at 3000 rpm at 4 °C and stored at −80 °C until further analysis.

### 4.4. Biochemical Analysis

The body weight and FBG of all rats were measured weekly throughout the experiment. Biochemical indices of triglyceride (TG), low density lipoprotein (LDL), high density lipoprotein (HDL), creatinine (CREA), and urea nitrogen (BUN) were determined by automatic biochemical analyzer.

### 4.5. Serum Sample Preparation and NMR Assay

Unfreeze the serum samples to room temperature during testing. The serum was centrifuged at 5000 rpm at 4 °C for 10 min. Then, 300 μL of supernatant, 180 μL of PBS (pH = 4.5), and 120 μL of TSP-containing D_2_O were added into a 5 mm NMR tube. The prepared samples were kept at 4 °C until NMR analysis.

The ^1^H-NMR spectra of all serums were obtained using a Bruker AVANCE III 500 MHz spectrometer at 298 K. Serum samples were recorded using the water-suppressed standard one-dimensional CPMG pulse sequence (recycle delay–90°(τ–180°–τ)n–acquisition) to obtain representative total metabolite compositions. Then, 128 transients were collected into 32k data points using a spectral width of 10 kHz with a relation delay of 3s, and the total echo time (2 nτ) was 100 ms [51].

### 4.6. Data Processing

All the obtained ^1^H-NMR spectra were manually phased and baseline corrected by MestReNova software (Mestrelab Research, Santiago de Compostella, Spain). The chemical shifts of the serum spectra were referenced to the lactate signal at δ1.324. Most of the substances in the spectrum had a good alignment effect, but there were still a small number of substances that could not be aligned. At this time, we re-calibrated these unaligned substances and then updated the new data to ensure the alignment effect of spectral peaks. The ^1^H-NMR spectra (δ 0.5ppm~9.5ppm) were divided into equal-width regions at an interval of 0.004ppm [51]. In order to eliminate the influence of water peak, the integral value of 4.67~5.22 PPM were set to 0. Then, normalization of the remaining spectral segments. All data were exported in “.txt” format and then imported into SIMCA-P 13.0 (Umetrics, Malmo, Sweden) for principal component analysis (PCA), partial least squares-discriminate analysis (PLS-DA), and orthogonal partial least squares discriminant analysis (OPLS-DA) models. Student’s t-test was used for statistical analysis to evaluate the significant differences of potential biomarkers, VIP > 1 and *p* value < 0.05 were selected as the potential biomarkers [52]. HMDB (http://www.hmdb.ca/) and KEGG (https://www.kegg.jp/) databases were used to identify potential biomarkers.

### 4.7. Pathway Enrichment Analysis of Biomarkers and Network Construction

MetaboAnalyst (http://www.metaboanalyst.ca) found and visualized the metabolic pathways of endogenous metabolites. As a result, the *p* values (*p* < 0.01) of pathways were regarded as potential therapy pathways in T2DM treated by HS.

We found targets that were related to potential pathways in T2DM treated by HS from the KEGG database. Finally, the network of potential biomarkers—metabolic pathways—targets was constructed, and the corresponding network analysis was conducted using Cytoscape 3.5.1 software (Cytoscape Consortium, San Diego, CA, USA).

### 4.8. Finding Chemical Composition of HS, Screening out Candidate Gene

In order to further comprehend the mechanism of HS on T2DM, the active compounds of HS were screened from the TCMSP database (http://lsp.nwu.edu.cn/tcmsp.php). Experience has shown that drugs that are in accordance with Lipinski’s “five rules” generally have great A (absorption) D (distribution) M (metabolism)E (excretion) properties. An oral bioavailability (OB) ≥ 30% is used to preliminary screening for drugs, and a drug likeness (DL) ≥ 0.18 is widely used to judge whether the phytochemical compounds have potential for drug development [53]. Therefore, in this study, compounds with Lipinski’s “five rules,” OB ≥ 30%, and DL ≥ 0.18 were selected as active compounds for further analysis. The TCMSP database provided the information about TCM ingredients and the targets about them [54]. The targets were manually normalized, and their official symbol in the Uniprot (http://www.uniprot.org/) database was determined. Through the above methods, we could obtain the relevant targets of the main chemical components of the HS. Finally, an active component-corresponding target interaction network could be constructed and displayed by Cytoscape software.

### 4.9. Combining Network Pharmacology and Metabonomics Analyses

The targets of relevant pathways obtained by metabolomics analysis were mapped with the HS-related targets obtained by network pharmacology analysis to find overlapping targets. These overlapping targets were considered as potential targets for the treatment of T2DM with HS, so that the active compounds corresponding to the targets in HS could be obtained, in turn, to elucidate the therapeutic mechanism of HS on T2DM.

## 5. Conclusions

In summary, by using ^1^H-NMR–based metabolomic technology, we found that there were 21 metabolites with significant changes in the model group. However, after treatment with HS, the disorder of these biomarkers can be reversed to varying degrees. These biomarkers were mainly involved in 14 metabolic pathways and 294 related targets. Network pharmacology identified 18 active compounds in HS, involving 135 targets. In addition, four targets (ACACA, MAOB, CAT, CA2) were the intersection of potential HS targets and potential biomarker targets, indicating that these four targets might be the most relevant targets for the treatment of T2DM with HS. In the next phase, we will verify the content change of the major target proteins obtained in this study, hoping to further clarify the pathogenesis of T2DM and the pharmacological mechanism of HS regulating T2DM.

## Figures and Tables

**Table 1:** Statistical analysis results of the main metabolites in serum.

No.	Metabolites	Chemical Shift(ppm)	VIP	Control/Model	Model/HS	Model/Metformin
1	lipid	0.85(m), 0.88(m), 1.57(m), 2.22(m), 1.26(m)	1.92	↑ *	↓ #	-
2	acetate	1.92(s)	1.01	↑ *	↓ #	↓ #
3	pyruvate	2.37(s)	2.87	↑ *	↓ #	↓ #
4	TMAO	3.27(s)	1.05	↑ *	↓ #	↓ ##
5	taurine	3.27(t), 3.43(t)	2.76	↓ *	↑ #	↑ #
6	glycine	3.54(s)	2.23	↓ **	↑ #	↑ ##
7	glycerol	3.54(dd), 3.66(dd)	1.41	↑ **	↓ #	↓ ##
8	isoleucine	0.93(t), 1.00(d), 1.96(m)	1.02	↑ **	↓ #	-
9	leucine	0.95(d), 0.97(d), 1.72(m), 3.65(dd)	1.06	↑ *	↓ #	-
10	valine	0.98(d), 1.03(d), 2.26(d), 3.60(d)	1.03	↑ **	↓ #	↓ #
11	glutamine	2.41 (m)	1.03	↓ **	↑ #	↑ ##
12	citrate	2.52(d), 2.67(d)	1.06	↑ *	↓ #	-
13	β-glucose	3.24(dd), 3.4(t), 3.46(ddd), 3.49(t),3.90(dd), 4.64(d)	1.75	↑ *	-	↓ #
14	α-glucose	3.53(dd), 3.72(dd), 3.76(dd), 3.83(ddd), 5.23(d)	2.89	↑ **	↓ #	↓ #
15	tyrosine	3.94, 6.89(d), 7.18(d)	1.04	↑ *	↓ #	↓ ##
16	3-hydrobutyrate	1.20(d), 2.31(dd), 2.41(dd)	2.37	↑ *	↓ #	↓ #
17	acetoacetate	2.28 (s)	1.52	↑ **	↓ #	↓ ##
18	succinate	2.40 (s)	1.22	↑ **	↓ #	↓ ##
19	unsaturated lipid	5.32 (m)	1.28	↑ *	↓ #	-
20	xanthine	7.75 (s)	1.01	↑ *	↓ *	↓ *
21	threonine	3.56(dd)	2.39	↑ **	↓ *	↓ *

Compared to control group: * *p* < 0.05; ** *p* < 0.01; compared with the model group # *p* < 0.05, ## *p* < 0.01.

**Table 2:** Results of pathway analysis using MetaboAnalyst database.

No.	Pathway Name	Total	Hits	*p*	Impact
1	Aminoacyl-tRNA biosynthesis	75	7	5.21 × 10^−7^	0
2	Valine, leucine and isoleucine biosynthesis	27	5	9.34 × 10^−7^	0.06148
3	Nitrogen metabolism	39	4	1.53 × 10^−4^	0
4	Valine, leucine and isoleucine degradation	40	4	1.69 × 10^−4^	0.02232
5	Citrate cycle (TCA cycle)	20	3	3.7 × 10^−4^	0.16797
6	Taurine and hypotaurine metabolism	20	3	3.7 × 10^−4^	0.35252
7	Alanine, aspartate and glutamate metabolism	24	3	6.45×10^−4^	0.20703
8	Glycolysis or Gluconeogenesis	31	3	0.0014	0.09576
9	Propanoate metabolism	35	3	0.00198	0.02982
10	Tyrosine metabolism	76	4	0.00201	0.04724
11	Butanoate metabolism	40	3	0.00292	0.1432
12	Phenylalanine metabolism	45	3	0.0041	0
13	Glycine, serine and threonine metabolism	48	3	0.00492	0.28435
14	Glyoxylate and dicarboxylate metabolism	50	3	0.00553	0.00326

The “Total” is the number of compounds in the pathway; the “Hits” represents the actual matched number from the user uploaded data.

## Supplementary Materials

The following are available online. Figure S1: Permutation test plots (200 permutations) for serum extracts: (A) Control group VS Model group; (B) HS group VS Model group. (C) Metformin group VS Model group, Table S1: The active compounds screened from Astragalus Radix and Dioscoreae Rhizoma by TCMSP database; Table S2: Potential target proteins of active compounds in Astragalus Radix and Dioscoreae Rhizoma predicted by TCMSP; Table S3: Pathways’ corresponding target proteins predicted by KEGG database; Table S4: Herb-active compound-target protein-pathway network of T2DM rats treated by Astragalus Radix and Dioscoreae Rhizoma.
